# Venezuela: out of the headlines but still in crisis

**DOI:** 10.2471/BLT.22.288269

**Published:** 2022-08-01

**Authors:** Shannon Doocy, Kathleen R Page, Charissa Liu, Hayley Hoaglund, Daniela C Rodríguez

**Affiliations:** a Johns Hopkins Bloomberg School of Public Health, 615 N. Wolfe St, Suite E8132, Baltimore, MD 21205, United States of America (USA).; b Johns Hopkins University School of Medicine, Baltimore, USA.

The Venezuelan political and economic crisis threatens the citizens’ health, though assessing its full impact is difficult due to lack of information. Available evidence shows a health system near collapse and the reversal of several decades of health gains.^1^ High levels of inflation and generalized poverty underscore the difficulties of maintaining health and adequate nutrition amidst the coronavirus disease 2019 (COVID-19) pandemic and rising global food insecurity.

In contrast to decreasing regional trends for Latin America and the Caribbean, under-five mortality in Venezuela has increased by an estimated 40% during the past decade. Current under-five mortality estimates are as high as 29 deaths per 1000 live births, with infants accounting for the majority of these deaths.^2^ At the general population level, noncommunicable diseases account for two thirds of deaths, followed by injuries, and communicable, maternal and perinatal conditions.^3^ Recent indications suggest a 3.5 year decrease in life expectancy compared to the previous generation.^4^

Government expenditure dropped by approximately one percentage point of GDP (gross domestic product) in the periods 2000–2010 and 2013–2019 and they remain more than one and a half percentage points below the Latin American and Caribbean region’s average.^5^ Water and electricity shortages, infrastructure decline and emigration of health personnel have led to a progressive loss of health system capacity. In March 2020, just before reporting the first local COVID-19 case, more than half of public hospital beds were inoperable and most laboratories were not fully operational. The pandemic exacerbated the situation, resulting in drastic reductions in non-COVID-19 bed capacity and surgical procedures.^6^ Access to health care and medicines remains limited due to health system capacity and supply chains, as well as high out-of-pocket expenses. In mid-2021, an estimated 18.8 million Venezuelans lacked access to health services, including 10.4 million with chronic diseases.^7^

Decreases in vaccination coverage have led to increased cases of measles, diphtheria and other vaccine-preventable diseases; measles re-emerged in a 2017–2019 outbreak of about 7000 confirmed cases. Despite recent vaccination campaigns, vaccination coverage is low and only half of infants are fully vaccinated for diphtheria–tetanus–pertussis and less than one third for measles.^5^ Malaria cases increased by nearly 900% between 2007 and 2017, and since 2017, Venezuela accounted for more than half of all malaria cases in South America.^8^ Underreporting is a concern for nearly all infectious diseases, including COVID-19. 

Access to adequate sexual and reproductive health services is limited. COVID-19 disruptions led to reductions of approximately 75% in skilled birth attendance, antenatal care and contraceptive use, which compares to regional declines averaging closer to approximately a third or less.^9^ Venezuela has the highest adolescent fertility rate in South America and fertility is projected to increase, despite rising numbers of women seeking abortions as a result of the scarce and costly nature of reproductive health services and contraceptives.^10^

Mental health is another major concern, as levels of anxiety and depression have increased and the crisis has curtailed mental health service capacity.^10^ However, the health system has limited to no capacity to care for patients with severe mental health disorders. Medication shortages have caused the deterioration of patients with previously well-controlled conditions, prompting The World Federation for Mental Health to declare a mental health crisis in the country.

Data on nutrition in Venezuela are scarce and national wasting estimates unavailable. A nongovernmental organization with nutrition programmes in multiple states found that 12% (5575/46 462) of children screened between 2017 and 2019 were wasted, with wasting rates almost double that figure in some states.^11^ Nutrition programme coverage is inadequate, with less than half of the target number of beneficiaries reached in many states in 2021, and rising numbers of consultations for undernutrition.^10^ Widespread efforts to ensure food security are critical given recent evidence of rising nutrition concerns and expectations that the global food crisis will make an adequate diet unaffordable for Venezuelans.

Some 14.3 million people in Venezuela need humanitarian assistance, the third most of any country in the world.^12^ Funding shortfalls for the United Nations appeal have translated to high levels of unmet needs. Further health sector investment is critical for addressing health needs and for mitigating the effects of the health system crisis. Given health worker shortages, investing in additional lower-level health workers who require less extensive training could expand access to basic health services, thereby allowing more qualified health providers, who are in short supply, to attend more complicated cases. To optimize effectiveness, health workers must have adequate supplies and medications to provide essential primary and preventive care. In primary care, health policy-makers should consider ensuring high coverage levels and service provision capacity in several areas, including maternal and child health and nutrition, sexual and reproductive health, noncommunicable diseases and mental health.
